# FGFBP1 as a potential biomarker predicting bacillus Calmette–Guérin response in bladder cancer

**DOI:** 10.3389/fimmu.2022.954836

**Published:** 2022-09-02

**Authors:** Fei Li, Henghui Zhang, Yu Wang, Zhihao Yao, Kunfeng Xie, Qixin Mo, Qin Fan, Lina Hou, Fan Deng, Wanlong Tan

**Affiliations:** ^1^ Department of Urology, Nanfang Hospital, Southern Medical University, Guangzhou, China; ^2^ School of Traditional Chinese Medicine, Southern Medical University, Guangzhou, China; ^3^ Department of Healthy Management, Nanfang Hospital, Southern Medical University, Guangzhou, China; ^4^ Department of Cell Biology, School of Basic Medical Science, Southern Medical University, Guangzhou, China

**Keywords:** bladder cancer, bacillus Calmette–Guérin, FGFBP1, biomarker, PD-L1

## Abstract

Accurate prediction of Bacillus Calmette–Guérin (BCG) response is essential to identify bladder cancer (BCa) patients most likely to respond sustainably, but no molecular marker predicting BCG response is available in clinical routine. Therefore, we first identified that fibroblast growth factor binding protein 1 (FGFBP1) was upregulated in failures of BCG therapy, and the increased FGFBP1 had a poor outcome for BCa patients in the E-MTAB-4321 and GSE19423 datasets. These different expression genes associated with FGFBP1 expression are mainly involved in neutrophil activation, neutrophil-mediated immunity, and tumor necrosis factor-mediated signal pathways in biological processes. A significant positive correlation was observed between FGFBP1 expression and regulatory T-cell (Treg) infiltration by the Spearman correlation test in the BCG cohort (*r* = 0.177) and The Cancer Genome Atlas (TCGA) cohort (*r* = 0.176), suggesting that FGFBP1 may influence the response of BCa patients to BCG immunotherapy through immune escape. Though FGFBP1 expression was positively correlated with the expressions of PD-L1, CTLA4, and PDCD1 in TCGA cohort, a strong association between FGFBP1 and PD-L1 expression was only detected in the BCG cohort (*r* = 0.750). Furthermore, elevated FGFBP1 was observed in BCa cell lines and tissues in comparison to corresponding normal controls by RT-qPCR, Western blotting, and immunohistochemical staining. Increased FGFBP1 was further detected in the failures than in the responders by immunohistochemical staining. Notably, FGFBP1 is positively associated with PD-L1 expression in BCa patients with BCG treatment. To sum up, FGFBP1 in BCa tissue could be identified as a promising biomarker for the accurate prediction of BCG response in BCa.

## Introduction

Bladder cancer (BCa) is one of the most common malignancies of the urinary tract worldwide, and it is projected to continue to rise in the next decade (1). At diagnosis, ~75% of bladder cancers are confined to the mucosa [nonmuscle invasive disease (NMIBCa)]. Transurethral resection of the bladder tumor (TURBT) combined with intravesical instillations is the mainstay therapy for those with NMIBCa. However, more than 50% of these cases will recur after resection and ~10% to ~20% will invade deeper layers (2). International guidelines recommend a clinical–pathological classification of NMIBC into low-, intermediate-, and high-risk groups. Further treatments aiming to reduce the risk of recurrence and/or progression into MIBC are warranted (3).

For many years, intravesical instillation of bacillus Calmette–Guérin (BCG) has been the gold standard treatment for patients with intermediate- or high-risk diseases to reduce the risk of recurrence and possibly progression (3–5). However, in approximately half of NMIBCa patients, intravesical BCG treatment fails due to BCG intolerance, BCG refractory to treatment, and BCG relapse (5). The current guidelines recommend early radical cystectomy with urinary diversion as a preferred option for those patients who would have a negative impact on their quality of life (6). Although various clinical and molecular biomarkers have been tested to help improve the accurate prediction of BCG response, currently, no ideal molecular biomarker predicting response to BCG therapy is available in clinical routine (4).

To find and identify ideal molecular biomarkers that can predict the response to BCG treatment in BCa, we identified that fibroblast growth factor binding protein 1 (FGFBP1) was upregulated in failures of BCG therapy, and the increased FGFBP1 had a poor outcome for BCa patients based on bioinformatics. Furthermore, FGFBP1 has been suggested to be involved in immune-related functions and pathways. FGFBP1 is a secretory protein that can specifically bind to fibroblast growth factors (FGFs) immobilized in the extracellular matrix to promote its release (7, 8). It has been receiving much more attention because of its considerable role in enhancing the biological and biochemical activities of FGFs and participating in the progression of several cancers (9–11). FGFBP1 was further demonstrated to be positive for PD-L1 expression in BCa tissues with BCG treatment. Briefly, FGFBP1 could be identified as a promising biomarker that may help to predict the prognosis of BCa patients with intravesical BCG treatment.

## Materials and methods

### Acquisition and preprocessing of datasets

Gene expression data and clinical information of BCa samples were obtained from The Cancer Genome Atlas Urothelial Bladder Carcinoma (TCGA-BLCA), the Gene Expression Omnibus (GSE19423, GSE163899, and GSE176178), and the ArrayExpress (E-MTAB-4321) databases. For each kilobase of an exon, we determined gene expression using fragments per million reads mapped (FPKM) in all the datasets. After effective normalization, the E-MTAB-4321, GSE19423, GSE163899, and GSE176178 datasets were subsequently integrated into the BCG cohort. All the patients received transurethral resection of BCa plus adjuvant BCG intravesical instillations. These patients were classified into BCG responders and failures based on the response to BCG therapy. BCG failures were defined as patients who had a recurrence (any stage or grade) of BCa within follow-up, and BCG responders had no recurrence during follow-up. The detailed clinical information of patients in each dataset is shown in **Table 1**.

**Table 1:** Clinical information of bladder cancer patients in BCG cohort.

Characteristics	ALL (*n* = 208)	E-MTAB-4321 (*n* = 88)	GSE19423 (*n* = 48)	GSE163899 (*n* = 32)	GSE176178 (*n* = 40)
**Age [years; no. of patients (%)]**					
≥65	126 (60.6)	53 (60.2)	28 (58.3)	19 (59.4)	26 (65)
<65	82 (39.4)	35 (30.8)	20 (41.7)	13 (40.6)	14 (35)
**Gender [no. of patients (%)]**					
Male	166 (79.8)	70 (79.5)	37 (77.1)	28 (87.5)	31 (77.5)
Female	42 (20.2)	18 (20.5)	11 (22.9)	4 (12.5)	9 (22.5)
**BCG [no. of patients (%)]**					
Responder	116 (55.8)	52 (59.1)	26 (54.2)	15 (42.9)	23 (57.5)
Failure	92 (44.2)	36 (40.9)	22 (45.8)	17 (57.1)	17 (42.5)
**Stage [no. of patients (%)]**					
CIS	2 (0.9)	2 (2.3)	0	0	0
Ta	52 (25)	52 (59.1)	0	0	0
T1	122 (58.7)	34 (38.6)	48	0	40 (100)
NA	32 (15.4)	0	0	32 (100)	0
**Grade [no. of patients (%)]**					
Low	93 (44.7)	40 (45.6)	28 (87.5)	25 (78.1)	0
High	75 (36.1)	48 (54.5)	20 (62.5)	7 (21.9)	0
NA	40 (19.2)	0	0	0	40 (100)

NA, not available.

### Identification of prognosis-related genes

The different expression gene (DEGs) analysis between BCG responders and failures in the E-MTAB-4321dataset was performed with the “limma” package (12). A *p* < 0.05 and |log2 (fold change)| ≥ 2.0 were regarded as significantly different. Univariate Cox regression analysis was used to identify DEGs with prognostic values. Kaplan–Meier survival curves were plotted to determine the prognostic value of the genes and compared by using the log-rank test. The receiver operating characteristic (ROC) curve was used to evaluate the accuracy of FGFBP1 for the prediction of BCG response.

### Gene set enrichment analysis

Gene set enrichment analysis (GSEA) was performed in the GSE19423 and E-MTAB-4321 datasets to gain insights into the biological pathways of the high- and low-expression groups stratified by FGFBP1 expression. A false discovery rate (FDR) of <0.25 and an adjusted *p* < 0.05 were considered statistically significant.

### Gene ontology and Kyoto Encyclopedia of Genes and Genomes pathway analysis of DEGs

The functions of DEGs were annotated using the Kyoto Encyclopedia of Genes and Genomes (KEGG) enrichment analysis and Gene Ontology (GO) analysis with the ‘‘Cluster Profiler’’ package (13).

### Estimation of tumor-microenvironment cell infiltration

The CIBERSORT algorithm was used to investigate the relative abundance of different immune cell types (14). The correlation between FGFBP1 and PD-L1 expression was examined using the Spearman correlation coefficient.

### Single-cell sequencing analysis

The Tumor Immunosingle Cell Centre (TISCH) database is used to analyze the expression of FGFBP1 at a single-cell level (15).

### Tissue specimens and cell line

Studies were done with the approval of the bioethics committee of Nanfang Hospital (Guangzhou, China). All subjects were informed and gave their written consent. All tissue specimens were obtained from patients diagnosed with BCa from the Chinese Han population at Nanfang Hospital from January 2018 to April 2022. A total of 15 pairs of tumor-paired tissues and normal adjacent tissues were obtained. Of these, 10 were male cases and five were female cases, with ages ranging from 36 to 68 years old.

The human BC cell lines UM-UC-3, T24, and SW780 were cultured in DMEM medium (Gibco, Carlsbad, CA, USA) supplemented with 10% fetal bovine serum (Serana, Berlin, Germany) and 1% penicillin/streptomycin at 37°C in a humidified incubator and a 5% CO_2_ atmosphere. The cell lines were authenticated by short tandem repeat (STR) profiling upon receipt and were propagated for <6 months after resuscitation.

### Real-time quantitative polymerase chain reaction

Real-time quantitative polymerase chain reaction (RT-qPCR) was performed as described in the previous study (16). The primer sequences used in this study are presented in **Supplementary Table S1**.

### Western blotting

The methods were described in our previous study (16). Briefly, rabbit monoclonal primary antibodies against human FGBP1 (dilution 1:1,000; Proteintech, Chicago, IL, USA) and glyceraldehyde-3-phosphate dehydrogenase (GAPDH) (dilution 1:1,000; Proteintech, Chicago, IL, USA) were used in this assay. The protein levels were normalized to those of GAPDH.

### Immunohistochemical staining

There were 17 clinical BCa sections in immunohistochemistry, including 10 responders and seven failures to BCG intravesical instillations. For immunohistochemical staining, the expressions of FGFBP1 and PD-L1 in tissue were examined by an ultrasensitive streptavidin-peroxidase (S-P) technique (Zhongshan Biotechnology Co. Ltd, Beijing, China) with the standard protocol as previously shown (17). Rabbit monoclonal primary antibodies against human FGBP1 (dilution 1:400; Proteintech, Chicago, IL, USA) and PD-L1 (dilution 1:200; Abcam, USA) were employed. Polyperoxidase rabbit IgG was used as the secondary antibody (Zhongshan Biotechnology Co. Ltd, China.). Negative controls were processed in an identical manner, with the primary antibody replaced by PBS. An independent assessment of immunoreactivity was conducted by two pathologists.

### Statistical analysis

R software (version 4.2.0, MathSoft, USA) was used for statistical analyses, and GraphPad Prism (version 9.0, GraphPad Software, USA) was used for graphing and analysis. Univariate Cox regression analysis was used to screen for genes with prognostic values. Survival analysis was performed by using the Kaplan–Meier method. Statistical analysis of RT-qPCR, Western blotting, and immunohistochemical staining was performed using two-tailed Student’s *t*-tests. *p* < 0.05 was regarded as statistically significant.

## Results

### Elevated FGFBP1 may be associated with the poor response to BCG treatment

**Figure 1:**
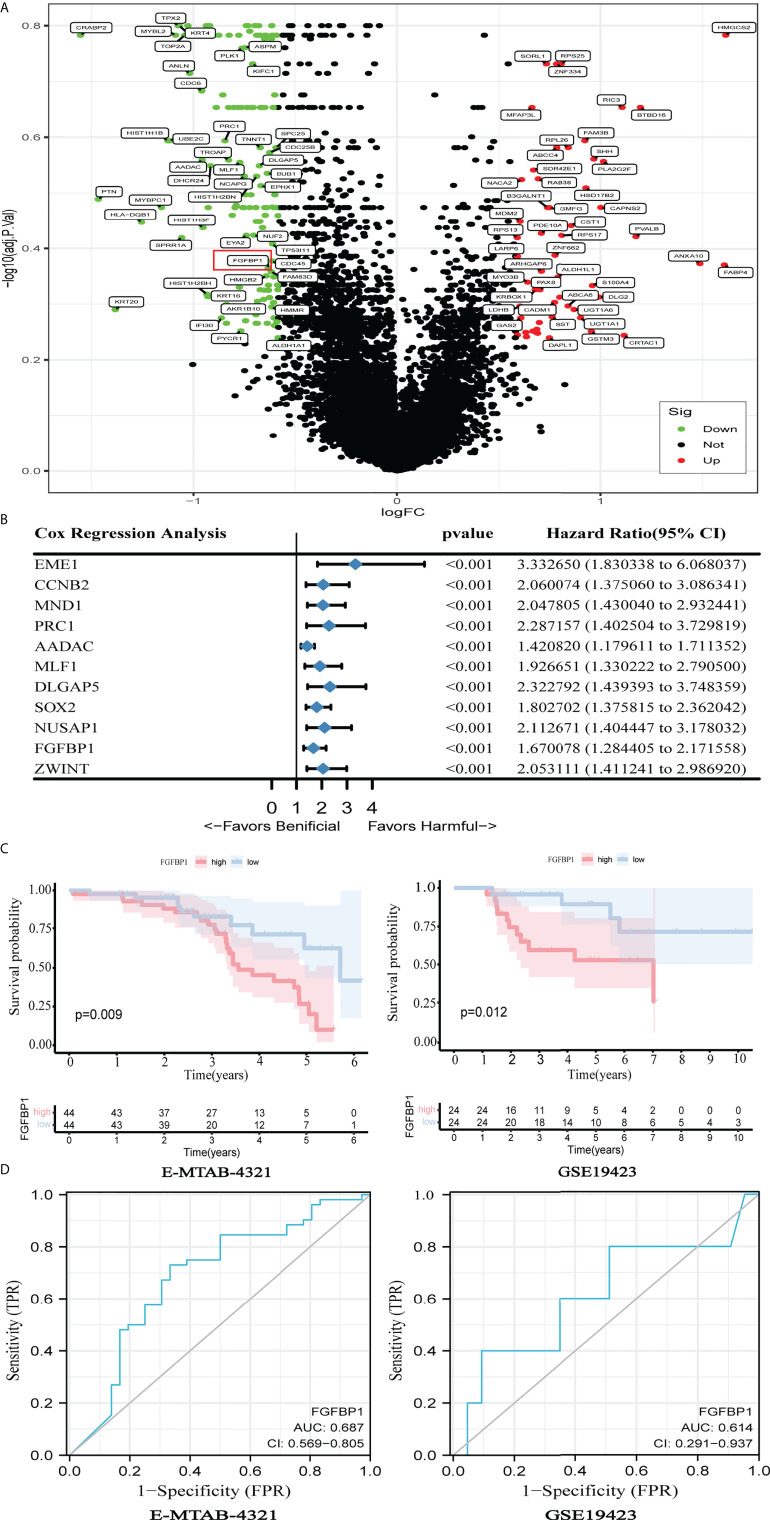
Elevated FGFBP1 may be associated with poor BCG response. **(A)** Volcano plot showed DEGs between BCG responders and failures in the E-MTAB-4321dataset, with FGFBP1 significantly upregulated in failures (marked in red). **(B)** Univariate Cox analysis revealed 11 genes that were associated with the prognosis of BCa in the E-MTAB-4321 dataset. **(C)** K-M survival curves indicated that elevated FGFBP1 expression was significantly associated with poor prognosis in the E-MTAB-4321 and GSE19423 datasets. **(D)** The ROC curves suggested that FGFBP1 has the ability to predict response to BCG treatment.

DEGs analysis was performed on the E-MTAB-4321 dataset. Of the detected DGEs, 80 were upregulated and 147 were downregulated genes (**Figure 1A**). The prognostic values of those DEGs were further calculated by using univariate Cox regression analysis with *p* < 0.001 as the screening criterion. Finally, 11 DEGs with prognostic values were found (**Figure 1B**). When the median of individual gene expression values was used as the cutoff points, patients in the E-MTAB-4321 and GSE19423 datasets were divided into low- and high-expression groups, respectively, to explore the relationship between gene expression and over survival. Noticeably, only FGFBP1 was significantly associated with patient prognosis in the E-MTAB-4321 and GSE19423 datasets (*p* < 0.05). The expression of FGFBP1 is negatively associated with the prognosis of BCa patients (**Figure 1C**; **Supplementary Figure S1A**). In addition, the role of elevated FGFBP1 expression in predicting the response to BCG in BCa patients was further explored using ROC curves. The results showed that elevated FGFBP1 may exhibit the ability to predict the response to BCG in the E-MTAB-4321 (AUC = 0.687) and GSE19423 (AUC = 0.614) datasets (**Figure 1D**). Furthermore, the result was confirmed in the GSE163899 and GSE176178 datasets (**Supplementary Figure S1B**). These findings suggested that elevated FGFBP1 may be associated with the poor response to BCG treatment.

### Potential biological functions of FGFBP1

**Figure 2:**
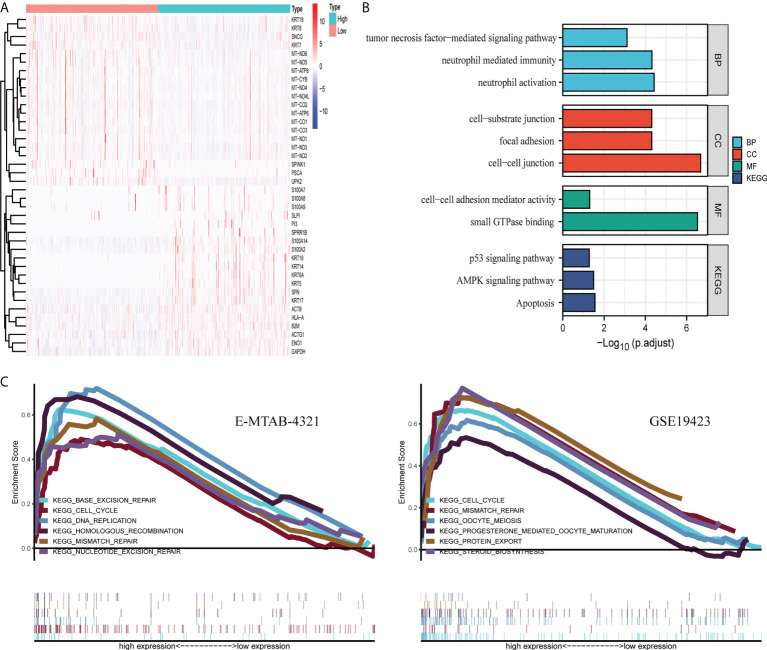
Potential biological functions of FGFBP1. **(A)** The heatmap of DEGs was defined by FGFBP1 in TCGA dataset, *p* < 0.05. **(B)** GO and KEGG analyses of DEG were performed in TCGA dataset. **(C)** GSEA analysis was conducted in high- and low-expression groups divided by the median value of FGFBP1 expression in the E-MTAB-4321 and GSE19432 datasets.

In TCGA-BLCA dataset, the median value of FGFBP1 expression was used as a cutoff for DEG analysis with an adjusted *p* < 0.01. As a result, 1,527 downregulated and 1,326 upregulated genes were found. The heatmap of those DEGs demonstrated the top 20 up- and downregulated genes (**Figure 2A**). GO and KEGG analyses were then performed on the DEGs to determine their biological functions. These DEGs associated with FGFBP1 expression were mainly involved in neutrophil activation, neutrophil-mediated immunity, and tumor necrosis factor-mediated signal pathways in biological processes. Genes associated with molecular function involve cell–cell linkage and focal adhesion. Among the cellular components, the main enrichment was the binding of small GTPases. KEGG analysis showed that DEGs were involved in 10 KEGG pathways, including the development of tumors, cellular regulation, the AMPK signaling pathway, and the p53 signaling pathway (**Figure 2B**). In addition, we performed a GSEA analysis in the E-MTAB-4321 and GSE19423 datasets with the median value of FGFBP1 expression as a cutoff. Both datasets were significantly enriched in the cell cycle as well as mismatch repair, suggesting a potential mechanism for FGFBP1 (**Figure 2C**).

### FGFBP1 was associated with tumor immune cell infiltration

**Figure 3:**
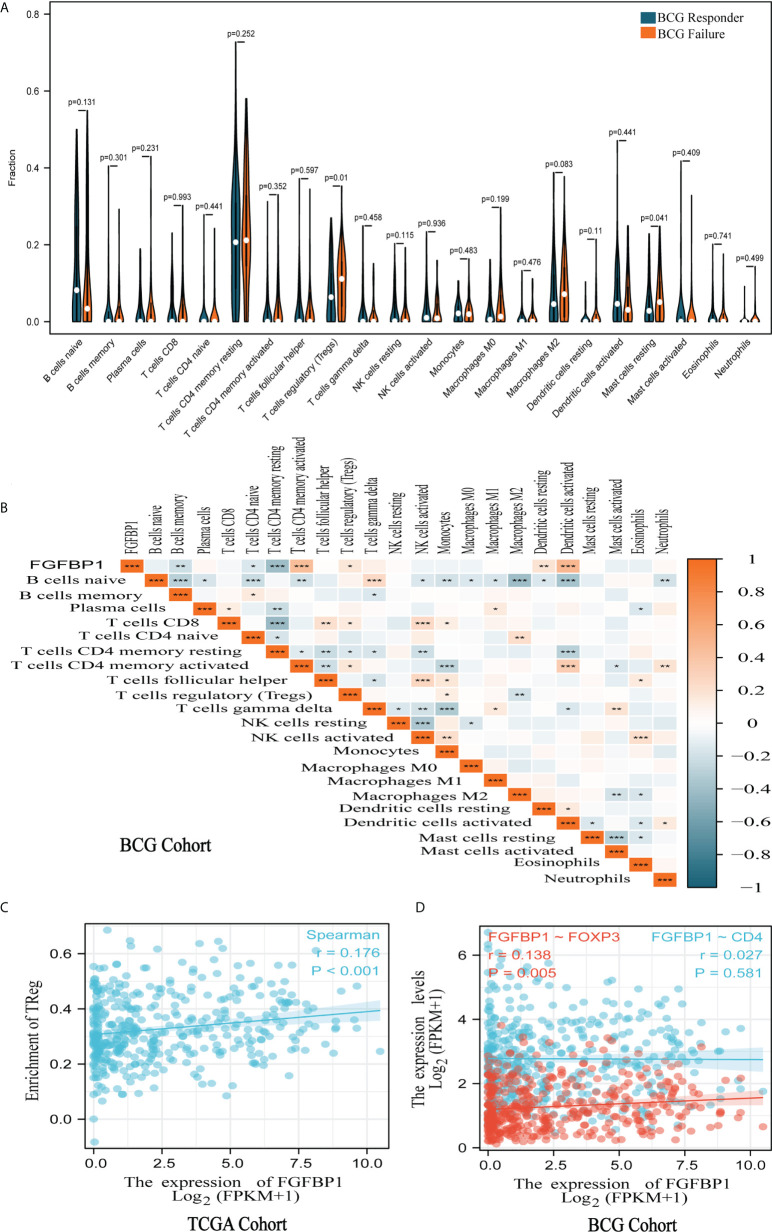
FGFBP1 was associated with tumor immune cell infiltration. **(A)** Violin plot of tumor immune cell infiltration showed that Tregs are significantly more infiltrated in the failures than in the responders in the BCG cohort. **(B)** A significant positive association was observed between FGFBP1 expression and Treg infiltration in the BCG cohort. **(C)** FGFBP1 is positively correlated with Treg infiltration in TCGA cohort. **(D)** FGFBP1 is positively correlated with FOXP3 expression in TCGA cohort. ^*^ *p* < 0.05; ^**^ *p* < 0.01; and ^***^ *p* < 0.001.

To further explore the relationship between FGFBP1 and the tumor immune microenvironment, the extent of immune cell infiltration in each sample of the BCG cohort was calculated using the CIBERSORT algorithm. The results showed that resting CD4^+^ T cells had the highest level of infiltration than other tumor immune cells in both BCG responders and failures (**Figure 3A**). Notably, Tregs had a significantly higher degree of infiltration in failures compared with responders in the BCG cohort (*p* = 0.01) (**Figure 3A**). Furthermore, there seems to be a potential correlation between FGFBP1 and Tregs, as clarified by the weak correlation coefficient (*r* = 0.177, *p* < 0.05) (**Figure 3B**). A similar result was found in TCGA cohort (*r* = 0.176, *p* < 0.001) (**Figure 3C**). There was a significant positive correlation between FGFBP1 and Foxp3 (*r* = 0.138, *p* = 0.005) (**Figure 3D**), which is a surface marker of Tregs. The results also showed a significant negative correlation between FGFBP1 expression and B-cell and CD4^+^ T-cell infiltration (*p* < 0.01) (**Figure 3B**). This indicated that FGFBP1 may influence the response of BCa patients to BCG intravesical instillations through immune escape.

### FGFBP1 is positively associated with PD-L1, as indicated by bioinformatic analysis

**Figure 4:**
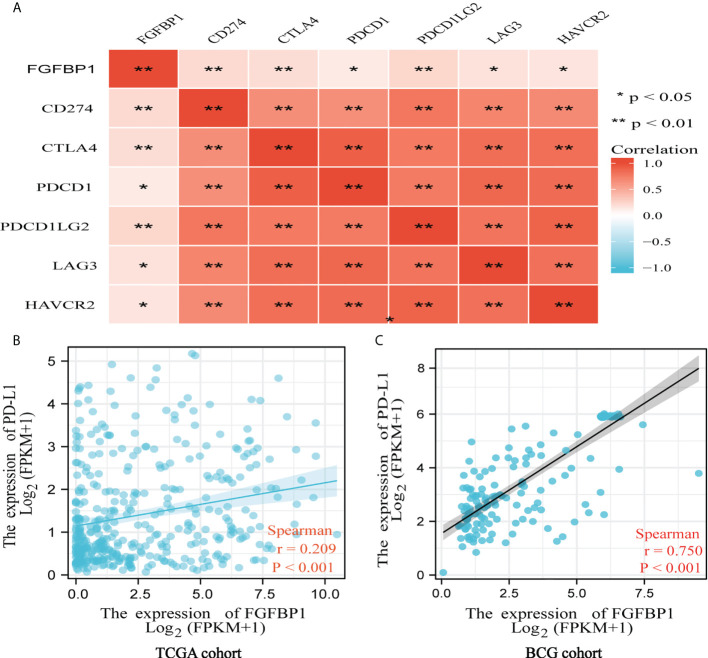
FGFBP1 is positively associated with PD-L1 indicated by bioinformatic analysis. **(A)** FGFBP1 was positively correlated with PD-L1, CTLA4, PDCD1, PDCD1LG2, LAG3, and HAVCR2. **(B)** FGFBP1 was positively associated with PD-L1 expression in TCGA cohort. **(C)** FGFBP1 was positively associated with PD-L1 expression in the BCG cohort.

The Spearman correlation coefficient was used to investigate the correlation between FGFBP1 and immune checkpoints. FGFBP1 expression was highly positively correlated with the expression of PD-L1, CTLA4, PDCD1, PDCD1LG2, LAG3, and HVACR2 in TCGA cohort (*p* < 0.01) (**Figure 4A**). Subsequently, the findings were confirmed in TCGA cohort (**Figure 4B**) and the BCG cohort (**Figure 4C**). Interestingly, a strong correlation between FGFBP1 and PD-L1 expression was found in the BCG cohort (*r* = 0.750, *p* < 0.001) (**Figure 4C**).

### Increased FGFBP1 is verified in BCa

**Figure 5:**
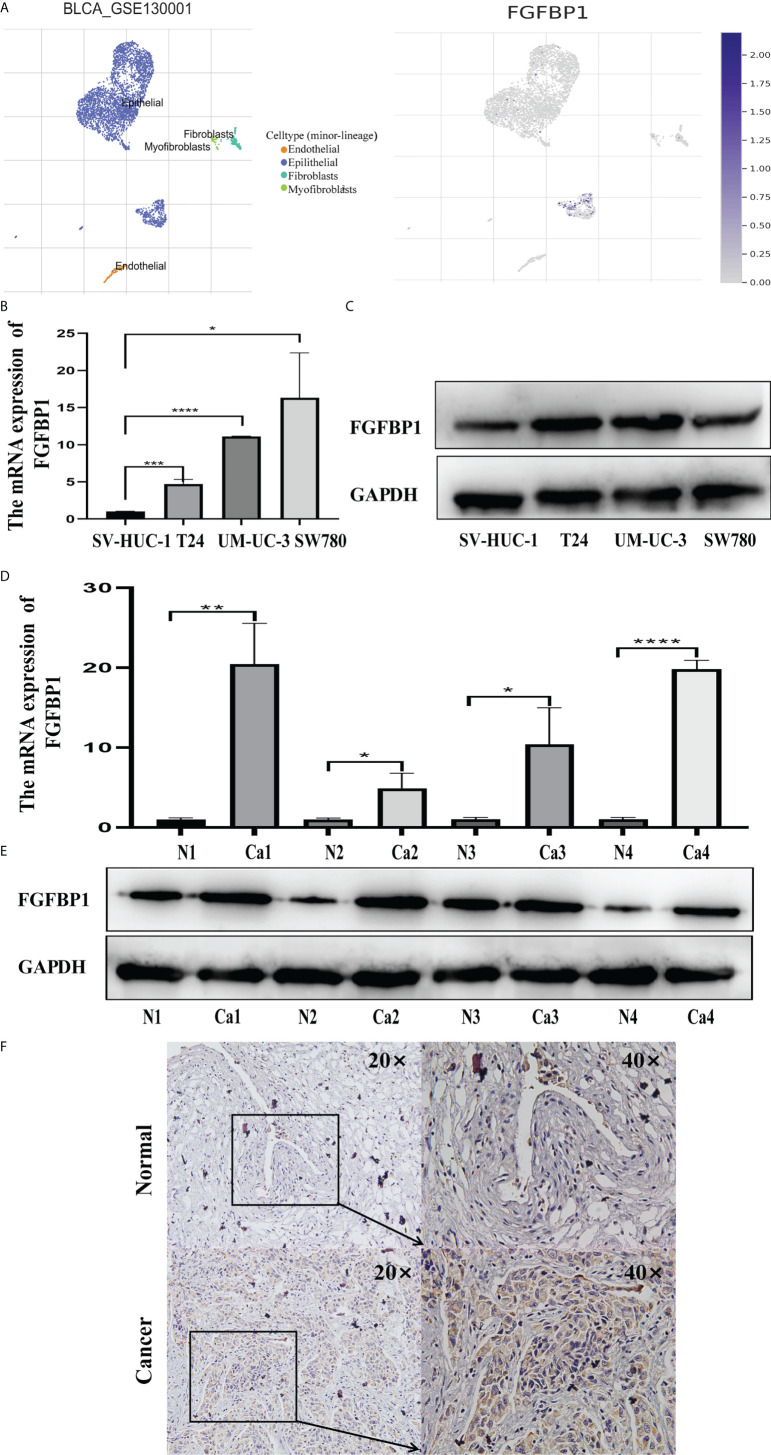
Increased FGFBP1 is verified in BCa. **(A)** Expression of FGFBP1 at the single-cell level in BCa using the TISCH database. Each dot corresponds to a single cell and is colored according to the cell cluster. The color density indicated the expression of FGFBP1. **(B)** FGFBP1 was verified in BCa cell lines by RT-qPCR. **(C)** FGFBP1 was verified in BCa cell lines by Western blotting. **(D)** FGFBP1 was verified in BCa tissues by RT-qPCR. **(E)** FGFBP1 was verified in BCa tissues by Western blotting. **(F)** Elevated FGFBP1 was observed in BCa tissues by immunohistochemistry staining. ^*^ *p* < 0.05; ^**^ *p* < 0.01; ^***^ *p* < 0.001, **** p < 0.0001.

We investigated the expression of FGFBP1 at the single-cell level in BCa using the TISCH database. FGFBP1 was found to be expressed mainly in BCa epithelial cells (**Figure 5A**). The expression of FGFBP1 was significantly elevated in BCa cell lines and BCa tissues in comparison to corresponding normal controls, as measured by RT-qPCR and Western blotting (**Figures 5B–E**). FGFBP1 protein was observed, apparently in the cytoplasmic compartments of cancerous cells by immunohistochemical staining (**Figure 5F**), which was consistent with our Western blotting results.

### FGFBP1 is positively correlated with PD-L1, as demonstrated in BCa

**Figure 6:**
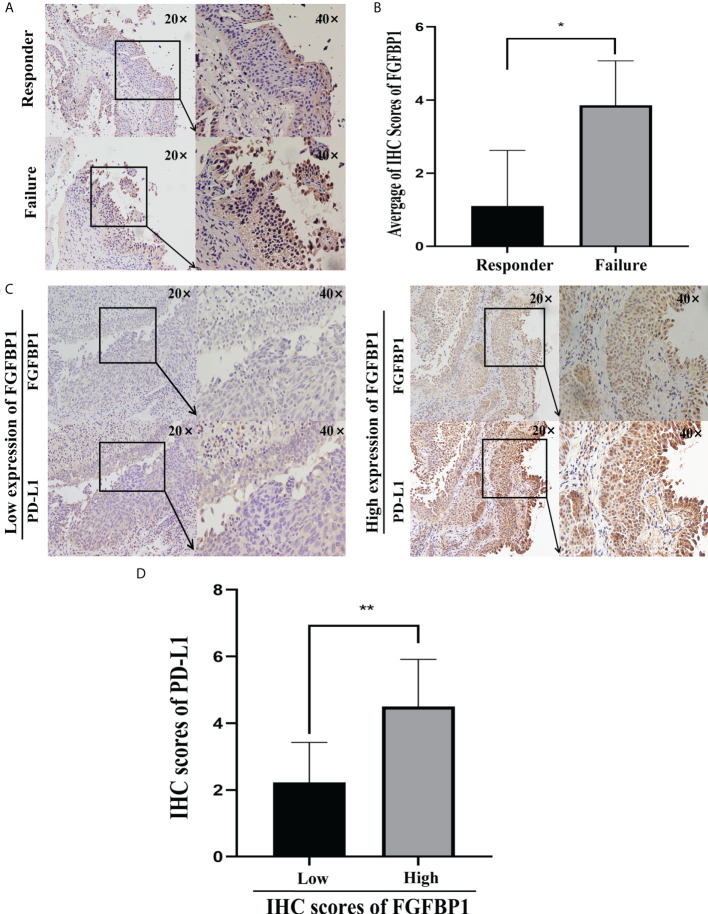
FGFBP1 is positively correlated with PD-L1 demonstrated in BCa. **(A)** FGFBP1 is elevated in failure compared with responders in BCa by immunohistochemical staining. **(B)** The immunohistochemical staining scores of FGFBP1 are higher in failures than responders in BCa. **(C)** The expression level of PD-L1 was significantly higher when FGFBP1 was increased by immunohistochemical staining when the BCG cohorts were divided into FGFBP1 high- and low-expression groups stratified by the median FGFBP1 expression. **(D)** The immunohistochemical staining scores of PD-L1 are positively associated with FGFBP1. ^*^ *p* < 0.05; ^**^ *p* < 0.01..

Expression of FGFBP1 was further detected by immunohistochemical staining in the BCG responders and failures. The staining of FGFBP1 protein in the failures was stronger than in the responders (**Figures 6A, B**). The immunohistochemical staining scores of FGFBP1 in each BCa tissue were calculated. The median score of FGFBP1 was 2.235, and we divided the cohorts into FGFBP1 high- and low-expression groups stratified by the median FGFBP1 expression. The expression level of PD-L1 was significantly higher (*p* < 0.001) when FGFBP1 was increased, implying that FGFBP1 may play significant antitumor immunity functions in failures (**Figures 6C, D**).

## Discussion

NMIBC is characterized by a high probability of recurrence and a risk of progression to muscle-invasive disease. NMIBC management requires a proper local resection followed by a risk-based treatment with intravesical agents (18). BCG intravesical adjuvant therapy has been effectively used in the management of intermediate- and high-risk NMIBC to prevent/delay tumor recurrence and/or progression (19, 20). However, 30% of those BCa patients would experience recurrence and progression into a more aggressive disease state (21). Accurate prediction of BCG response is essential to identify the patients most likely to respond sustainably, but no molecular marker predicting BCG response is available up to date.

In this study, we initially identified that BCa patients with higher FGFBP1 expression had a worse prognosis in the E-MTAB-4321 and GSE19423 datasets. Furthermore, ROC curves indicated that elevated FGFBP1 may exhibit the ability to predict the response to BCG in the E-MTAB-4321 (AUC = 0.687) and GSE19423 (AUC = 0.614) datasets. Moreover, the expression of FGFBP1 is significantly elevated in failures compared with responders to BCG treatment, as confirmed by Western blotting and immunohistochemical staining. Briefly, FGFBP1 could predict the response to BCG for BCa patients. However, the roles of higher FGFBP1 in failures have not been studied extensively.

Although the mechanism concerning BCG action is still not completely understood, one main explanation is that BCG exposure to urothelium and bladder-resident macrophages elicits an inflammatory and immune response against tumoral cells (22–26). Moreover, an intrinsic or acquired immune resistance would be the possible resistance mechanism to BCG treatment. It is known that PD-L1 overexpressed in cancer cells could let those cells evade the immune response, inducing T-cell anergy (24, 27, 28). Immunotherapy has become an increasingly promising therapeutic method for advanced BCa, with PD-L1 inhibitors being able to halt immune evasion of cancer cells by preventing PD-1 from binding to its ligand (28).

FGFBP1, belonging to the FGFBP family, is a secretory protein that can specifically bind to FGFs immobilized in the extracellular matrix and present them to their cognate receptors (8). FGFBP1 is expressed in epithelial cells in the skin, eye, ileum, and colon (8, 29–31), and plays an important role in proliferation and differentiation during embryonic development and wound healing (30, 32). Moreover, it was also found to be upregulated in various cancers than its low expression in normal adult tissues (30). Elevated FGFBP1 facilitates cancer growth and metastasis, which was demonstrated to act as an angiogenic switch molecule in cancer by enhancing FGF signaling including angiogenesis during cancer progression (33, 34). FGFBP1 was reported to be regulated by different transcription factors, including β-catenin/TCF4, C/EBP, and KLF5. Correspondingly, Wnt/β-catenin and KLF5-induced tumorigenesis and metastasis are decreased after FGFBP1 downregulation (7, 35, 36). However, the mechanisms of elevated FGFBP1 in failures of BCG treatments in BCa patients have not been known.

Chun et al. observed that a lower baseline infiltration level of Treg predicted a better response to BCG treatment (37). In our study, the CIBERSORT algorithm estimating immune cell infiltration indicated that there was a higher degree of Treg infiltration in failures compared to responders in the BCG cohort (*p* = 0.01). Interestingly, the expression level of FGFBP1 is positively correlated with Treg infiltration by the Spearman correlation test, suggesting a potential mechanism of action for FGFBP to BCG response in BCa patients. Furthermore, a strong correlation was observed between FGFBP1 and PD-L1 expression in the BCG cohort (*r* = 0.750, *p* < 0.001). Unexpectedly, the expression levels of FGFBP1 and PD-L1 were also found to be significantly higher in failures compared with responders by Western blotting and immunohistochemical staining analyses.

The mechanism underlying the higher FGFBP1 in failures of BCG treatment should be further explored. One possible explanation is that the increased FGFBP1 was positively associated with PD-L1 in BCa cells, which may cause BCa patients to evade the immune response when they receive BCG treatment. Another possibility might be the important role of FGFBP1 in tumor angiogenesis and cancer progression. A better understanding of the novel mechanisms may yield new knowledge for therapeutic purposes.

Several important strengths should be noted in our study. We first observed that FGFBP1 is highly expressed in BCa tissues in failures compared with responders to BCG treatment and that high expression of FGFBP1 is associated with a poor outcome for BCa patients based on the E-MTAB-4321 and GSE19423 datasets. Our bioinformatics also found that the DEGs identified by FGFBP1 were enriched in immune-related functions and pathways. Mechanistically, increased FGFBP1 may be positively associated with the upregulation of PD-L1 in a dependent manner in BCa patients with BCG treatment. Collectively, our results provide a promising biomarker for predicting response to BCG therapy in BCa patients.

Some limitations need to be taken into account when FGFBP1 is used for screening responses to BCG therapy. A limitation of the study is that FGFBP1 and PD-L1 were verified in a small number of BCa patients with BCG intravesical adjuvant therapy. The results require verification in larger sample sets, including enough follow-up time and detailed clinical information. Furthermore, increased FGFBP1 and PD-L1 were found in the BCG failure cohort on the basis of bioinformatics and experiments. The explanation may be the immune escape in failures caused by high expression of FGFBP1. However, the roles of elevated FGFBP1 in failures are needed to be further elaborated.

To sum up, the expression level of FGFBP1 is shown to be significantly upregulated in failures compared with responders. Our study thus indicates that FGFBP1 in BCa tissue may be a potential molecular biomarker for the accurate prediction of BCG response in BCa. Further research is warranted to investigate its putative mechanistic roles in the pathogenesis of BCa with intravesical BCG treatment.

## Data availability statement

The original contributions presented in the study are included in the article/**Supplementary Material**. Further inquiries can be directed to the corresponding authors.

## Ethics statement

This study was reviewed and approved by the bioethics committee of Nanfang hospital (Guangzhou, China). The patients/participants provided their written informed consent to participate in this study. Written informed consent was obtained from the individual(s) for the publication of any potentially identifiable images or data included in this article.

## Author contributions

FL and HZ performed and conceived the study. FL, LH, and WT wrote the manuscript. KX and QM provided clinical information. FL, HZ, YW, and ZY were responsible for collecting and analyzing public data, completing experiments, and drawing charts. FL, QF, and WT assisted in improving the quality of language. FL, FD, and WT completed the final revision of the manuscript. All authors contributed to the article and approved the submitted version.

## Funding

This study was supported by the Natural Science Foundation Committee of China (NSFC 82073162) (WT), the Natural Science Foundation of Guangdong Province of China (2021A1515010762) (FL), the Outstanding Youth Development Scheme of Nanfang Hospital, Southern Medical University (2019J009) (FL), the Dean’s research fund of Nanfang Hospital, the Southern Medical University (2020Z005) (FL), (2019B008) (LH), and the Beijing Bethune Charitable Foundation (mnzl202017) (FL).

## Conflict of interest

The authors declare that the research was conducted in the absence of any commercial or financial relationships that could be construed as a potential conflict of interest.

## Publisher’s note

All claims expressed in this article are solely those of the authors and do not necessarily represent those of their affiliated organizations, or those of the publisher, the editors and the reviewers. Any product that may be evaluated in this article, or claim that may be made by its manufacturer, is not guaranteed or endorsed by the publisher.
