# Supplementary material for: FGFBP1 as a potential biomarker predicting bacillus Calmette–Guérin response in bladder cancer
Source: Front Immunol. 2022 Sep 2;13:954836. doi: 10.3389/fimmu.2022.954836 (PMC9478507; doi:10.3389/fimmu.2022.954836)
Supplement: Supplementary file 1 [file DataSheet_1.docx]

Supplementary Material

# Supplementary Figures and Tables

## Supplementary Figures


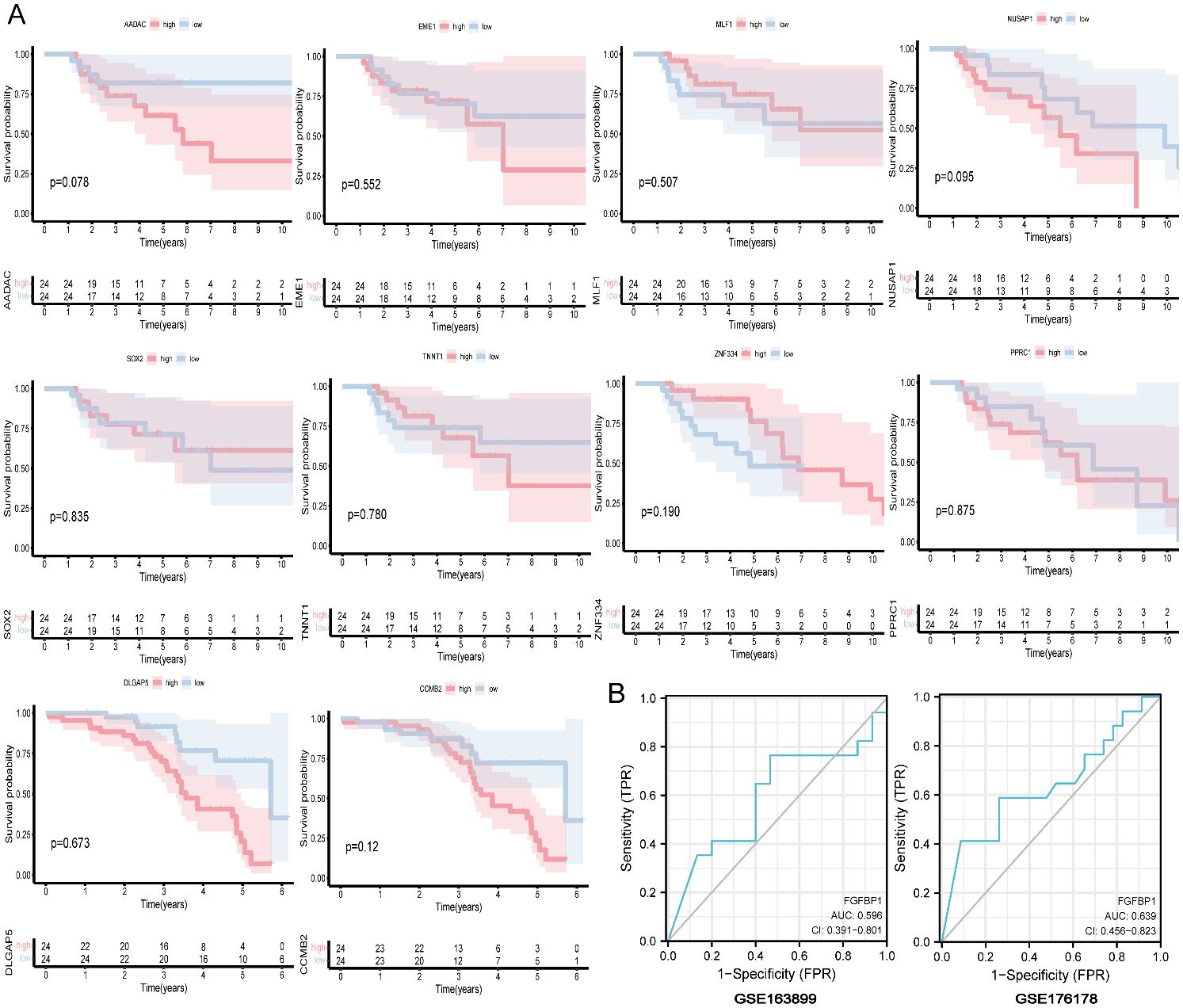


**Supplementary Figure 1.** (A) K-M survival curves for individual genes in GSE19423 datasets. (B) the ROC curves of GSE163899 and GSE176178 datasets indicate FGFBP1 has the ability to predict response to BCG treatment

## Supplementary Tables

**Supplementary Tables 1 Primers sequence.**

| **Genes** | **Forward** | **Reverse** |
| --- | --- | --- |
| FGFBP1 | CGTGTGCTCAGAACAAGGTG | GAGCAGGGTGAGGCTACAGA |
| β-Actin | CTCCCTGGAGAAGAGCTACGAGC | CCAGGAAGGAAGGCTGGAAGAG |
